# Trends in management and healthcare resource utilization for achalasia following adoption of per-oral endoscopic myotomy

**DOI:** 10.1007/s00464-025-12524-4

**Published:** 2026-01-05

**Authors:** Michael Rosenheck, Itegbemie Obaitan, Neil Sharma, Mohammad Al-Haddad, John M. DeWitt, IU-Parkview Achalasia Working Group, M. Rosenheck, I. Obaitan, V. Rajagopalan, J. Montrose, H. Baydoun, A. Al Qady, A. Hajj-Ali, M. G. Batarseh, Z. Javed, A. S. Dahbour, U. Pamidimukkala, O. Momoh, S. Zelt, A. Perkins, N. Sharma, M. A. Al-Haddad, J. M. DeWitt

**Affiliations:** 1 Department of Internal Medicine, Indiana University Health, Indianapolis, IN USA; 2 Division of Gastroenterology and Hepatology, Indiana University Health, 550 North University Blvd, UH 4100, Indianapolis, IN 46202 USA; 3 Parkview Research Center, Parkview Regional Medical Center, Fort Wayne, IN USA

**Keywords:** Achalasia, Per-oral endoscopic myotomy, Heller myotomy, Pneumatic dilation

## Abstract

**Background:**

Per-oral endoscopic myotomy (POEM), Heller myotomy (HM), and pneumatic dilation (PD) offer definitive interventions of achalasia, but comparative analysis of long-term healthcare resource utilization is not well described. This study explores longitudinal trends and one-year post-procedure healthcare resource utilization in Indiana following local adoption of POEM in 2014.

**Methods:**

This multicenter retrospective statewide cohort study utilized relevant ICD-9, ICD-10, and CPT codes to identify all patients with achalasia who underwent definitive index disease intervention with POEM, HM (laparoscopic HM alone or LHM with intraoperative conversion to open myotomy) or PD between January 2008 and November 2022 with ≥ 1 year follow-up. Multivariate logistic regression was utilized to determine post-intervention healthcare resource utilization. Temporizing interventions were defined as < 30 mm balloon dilation or Botox injections.

**Results:**

621 patients who underwent POEM (*n *= 363, mean age 54.18 years, 39% female), HM (*n* = 246, mean age: 48.71 years, 47% female), and PD (*n *= 12, mean age 52.75 years, 50% female) from two tertiary centers in Indiana were identified. From 2014 to 2022, the use of POEM increased 31-fold, while the use of HM decreased 19-fold between 2008 and 2022. Within one year of treatment, POEM was associated with less definitive reintervention compared to HM (OR 0.10, 95% CI 0.01–0.62, *p* = 0.01) or PD (OR 0.002, 95% CI 0.0001–0.023, *p* < 0.001) but more diagnostic testing than HM (OR 20.9, 95%CI 10.8, 40.4, *p* < 0.001). Hospital utilization within 30 days, temporizing interventions within one year, and the frequency of adverse events were similar.

**Conclusions:**

During the study period, use of POEM increased 31-fold while use of HM decreased 19-fold. POEM was associated with increased healthcare resource utilization, more post-procedure testing, lower odds of definitive disease reintervention, and similar rates of adverse events compared to HM and PD.

Achalasia is a rare disorder of esophageal motility characterized by aperistalsis and increased lower esophageal sphincter tone. It is prevalent in about 1–2 per 100,000 people, occurs without a clear predominance of gender or race, and is most common between 30 and 60 years of age [1]. Related symptoms of dysphagia and regurgitation may lead to weight loss, significant debility, and failure to thrive[1]. Definitive treatment involves disruption of the lower esophageal sphincter (LES) which has historically been achieved either endoscopically with pneumatic dilation (PD) or surgically by open (OHM) or Heller myotomy (HM). Due to its efficacy, availability, and durability, HM has been the most frequently used definitive procedure for treatment of achalasia for decades in the United States. In fact, evaluation of the Nationwide Inpatient Sample (NIS) from 1992 to 2011 found that the number of HM procedures increased from 1576 to 5046 cases during the study period [2].

Per-oral endoscopic myotomy (POEM) is a flexible endoscopic procedure first described in 2010 [3] that incises the LES similar to HM but is performed without a concomitant fundoplication. Randomized trials demonstrate the clinical efficacy of POEM is superior to PD [4] and equivalent to HM [5]. Due to the novelty and demonstrated efficacy of POEM, recent studies have evaluated temporal trends in treatment of achalasia. Evaluation of the NIS from 2016 to 2020 found that hospital admissions for achalasia following POEM increased from 6 to 10%, while those following HM decreased from 49 to 41% [6]. Furthermore, a retrospective study of a United States commercial insurance database found that treatment of achalasia between 2010 and 2017 in 1921 patients was associated with a 19-fold increase in use of POEM, whereas use of laparoscopic Heller myotomy (LHM) and PD decreased during the study period [7]. This study also found that POEM led to higher healthcare resource utilization (HRU) compared to LHM, but lower healthcare resource utilization and higher costs compared to PD. However, these studies [6, 7] are limited by use of anonymized medical records and therefore inability to track individualized information for reported adverse events and clinical outcomes. Identified data from healthcare records in local regions would be helpful to supplement these data from national databases on recent trends of definitive therapy techniques for achalasia.

The purpose of our statewide multicenter study was to evaluate temporal trends in the adoption and use of POEM relative to HM and PD, and to compare the short- and medium-term HRU and procedure-related adverse events among all three techniques. We hypothesized that similar to previously reported anonymized national insurance database studies, our statewide use of POEM during the study period would increase at the expense of increased HRU compared to HM.

## Materials and methods

This was a retrospective cohort study of achalasia patients 13 years or older in the state of Indiana who underwent an index definitive treatment with POEM, HM (either laparoscopic or laparoscopic with conversion to open myotomy) or PD between January 2008 and November 2022. All patients had at least 12 months of follow-up after intervention. The diagnosis and subtype of achalasia were made by esophageal manometry when available. When manometry was not available, achalasia was confirmed by delayed esophageal emptying on timed barium esophagram. Patients with a non-achalasia motility disorder, planned open Heller myotomy, prior esophageal or gastric surgeries, prior POEM, PD or HM or a known pre-procedural diagnosis of esophageal or gastric malignancies were excluded.

We specifically excluded pre-planned open heller myotomy cases as the laparoscopic approach has been shown to have superior short-term and similar long-term outcomes compared to open heller myotomy[8]. We did not however exclude LHM cases with intraoperative conversion to open myotomy, as this is a not uncommon real-world clinical scenario. The decision was made to also include PD as a comparator group despite POEM and LHM being considered the current standard of care treatment strategies because PD is still routinely used in a few clinical practices nationally. Data from the pneumatic dilation cohort may therefore still have some value to interested clinicians. Other studies assessing trends in achalasia treatment strategies routinely include data from all three modalities.

Patients were screened from the Indiana Health Information Exchange (IHIE), a statewide database of prospectively collected data that captures 98% of all healthcare data within the state of Indiana. Patients with the diagnosis of achalasia were identified using the ICD9 code 530.0 and ICD10 code K22.0. Procedures performed were identified by using the Current Procedural Terminology (CPT) codes for LHM (CPT 43279), PD (CPT 43458, 43,214 or 43,233) and POEM (43,499 and 43,497). Since the current CPT code 43,497 for POEM was not approved until Jan 1, 2022, the CPT code used to bill POEM during the study period was usually an unlisted esophageal code (43,499). Therefore, patients undergoing POEM were identified by prospective institutional databases which commenced with the onset of the initial POEM procedure at each study location. Our search of the IHIE database found that all patients who received one of the three procedures for index achalasia intervention during the study period were treated at only two large, tertiary referral health systems (Indiana University Health in Indianapolis and Parkview Health in Fort Wayne). This study was approved, and informed consent was waived by the institutional review boards (IRBs) at both Indiana University Health and Parkview Health. After identification, patients were grouped into those with an index intervention of POEM, HM (defined in our study specifically as patients who underwent LHM or had intraoperative conversion from LHM to open HM at the discretion of the surgeon), and PD as specified above. Identified medical records including assigned sex and self-reported gender were reviewed for variables of interest as outlined below were extracted for analysis.

### Primary outcomes

The primary outcomes tracked for this study included:

#### Temporal trend of achalasia technique utilization

An analysis of the year-over-year utilization of each of the three treatment techniques for achalasia during the study period to gauge the incidence of statewide adoption of POEM relative to the other techniques.

#### Post-intervention healthcare resource utilization

We assessed healthcare resource utilization via four metrics:

- Diagnostic testing within one year of intervention. Diagnostic testing was considered use of esophagram, esophagogastroduodenoscopy (EGD) or chest computed tomography (CT) scan to evaluate the previous achalasia intervention, esophageal manometry or endoscopic functional lumen imaging probe (EndoFLIP). At the onset of procedure adoption, post-POEM management at both hospitals was driven by institutional protocols requiring post-POEM EGD, FLIP, pH study, and manometry between 6 and 12 months after POEM. However, as proceduralists became more experienced and comfortable with post-procedure patient care, each transitioned to only requiring a post-POEM EGD with FLIP for all patients with pH studies and manometries performed only when required clinically. Use of diagnostic testing was further stratified into early (within 30 days) and delayed (medium- to long-term) (31 days to 1-year) post-intervention testing.
- Procedure-related, post-discharge hospital utilization within 30 days of intervention. This was defined as any emergency department visit, inpatient admission or intensive care unit (ICU) stay following hospital discharge for achalasia intervention.
- Temporizing reintervention for achalasia performed within one year, defined as use of botulinum toxin (Botox) injections or small balloon (< 30 mm) dilation of the LES.
- Definitive reintervention for achalasia, defined as use of POEM, HM or PD, within one year following index intervention.

### Secondary outcomes

#### Safety

Data on relevant procedure-related adverse events (AEs) including bleeding requiring ≥ 1 blood transfusion, bowel perforation, pneumothorax requiring intervention, myocardial infarction, cerebrovascular event, infections, respiratory dysfunction (requiring either significant oxygen supplementation defined as > 2L of oxygen via nasal cannula and/or intervention) and death were recorded. Severity of AEs was graded with the Clavien–Dindo scale[9] for HM and the Adverse Events in Gastrointestinal Endoscopy (AGREE) scale[10] for endoscopic interventions (POEM and PD).

#### Data analysis

A descriptive analysis of baseline demographics, achalasia interventions, Charlson Comorbidity Index (CCI), AEs, post-procedure testing data, and reintervention data (both temporal and definitive) was performed. A non-parametric test of trends was used to evaluate the changes in use of all techniques over the study period. Means with standard deviation (SDs) for continuous variables and values with percentages were reported for categorical variables. For the primary analysis comparing the three techniques, an analysis of variance (ANOVA) was used to compare continuous outcomes by intervention type, while Fisher’s exact test was used to compare categorical variables by intervention type. Multivariate logistic regression was used to determine the association between intervention with hospital utilization within 30 days of the index intervention, utilization of diagnostic testing, and temporizing procedures within a year of index procedure and definitive achalasia reintervention adjusting for age, CCI, and year of intervention. Finally, we excluded the small PD cohort and performed a sensitivity analysis using multivariate logistic regression modeling to directly compare the various metrics of HRU between the POEM and HM groups. Two-sided p values < 0.05 were considered statistically significant. Data analysis was carried out using SAS v9.4 (SAS Institute, Cary NC).

## Results

### Study population

**Fig. 1:**
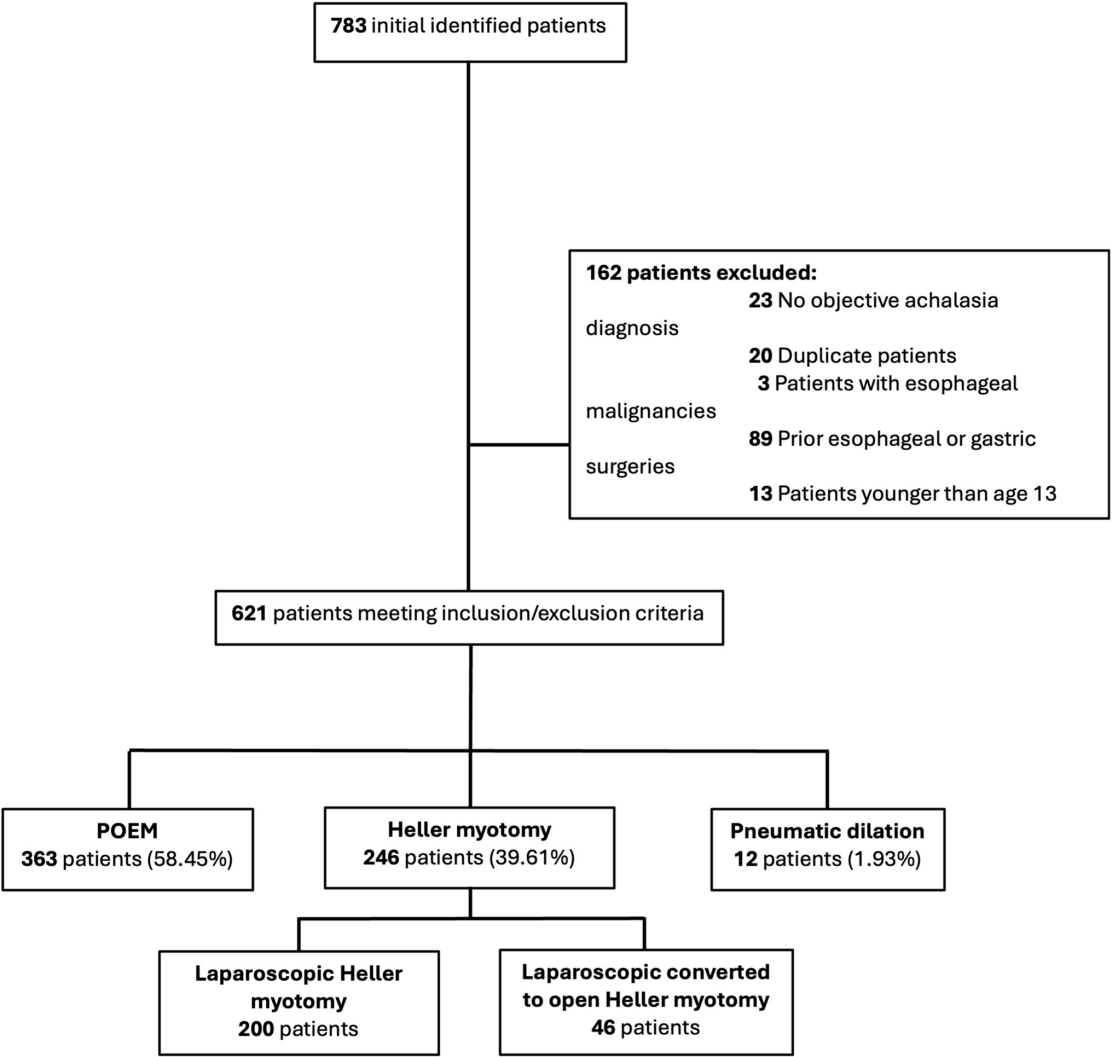
Study flow diagram of patients included in final analysis

We identified 783 patients with a diagnosis of achalasia who underwent index disease treatment with HM (specifically LHM or planned LHM with intraoperative conversion to open myotomy), PD, or POEM during the study period (Fig. 1). Of these patients, 162 were excluded as outlined in Fig. 1. The study population included 621 patients who underwent POEM (*n* = 363, 58%), HM (*n* = 246, 40%), and PD (*n* = 12, 2%). Forty-six (19%) of the 246 planned LHM interventions required intraoperative conversion to open surgical myotomy.

### Patient characteristics

Among the study population, the HM group was younger (*p* = 0.002) and more likely have pre-procedure esophagram (*p *= 0.001) compared to the other two groups (Table 1). The POEM group was more likely to undergo preoperative EGD (*p* = 0.001), esophageal manometry (*p* < 0.001), and EndoFLIP (*p* < 0.001). HM (58.13%) and PD (83.33%) patients were more likely to have an unknown or undocumented achalasia subtype compared to the POEM group (6.06%, *p* < 0.001). The PD group had a higher proportion of patients with CCI scores > 6 (25%) compared to the POEM (5.79%) and HM (4.07%) groups. A CCI of 0–3 was found more frequently in the HM group (83.74%) compared to the PD (66.67%) or POEM group (71.63%, *p* = 0.001).

**Table 1:** Patient characteristics

	Heller myotomy (*n* = 246)	Pneumatic Dilation (*n* = 12)	POEM (*n* = 363)	p value
Demographics				
Mean Age (SD)	48.71 (18.73)	52.75 (17.73)	54.18 (18.61)	0.002
Female, n (%)	116 (47.15)	6 (50.00)	141 (38.84)	0.101
Ethnicity, n (%)				0.089
White	199 (82.57)	9 (75.00)	306 (87.93)	
Black	30 (12.45)	2 (16.67)	35 (10.06)	
Other	12 (4.98)	1 (8.33)	7 (2.01)	
Achalasia diagnosis method				
Esophagram	131 (53.25)	5 (41.67)	139 (38.29)	0.001
Upper endoscopy	197 (80.08)	10 (83.33)	328 (90.36)	0.001
Esophageal manometry	213 (86.59)	7 (58.33)	342 (94.21)	< 0.001
EndoFLIP	9 (3.66)	2 (16.67)	195 (53.72)	< 0.001
Other modalities	7 (2.85)	1 (8.33)	33 (9.09)	0.004
Achalasia subtype, n (%). < 0.001				
Type 1	18 (7.32)	0 (0)	49 (13.50)	
Type 2	77 (31.30)	2 (16.67)	252 (69.42)	
Type 3	8 (3.25)	0 (0)	40 (11.02)	
Unknown/undocumented	143 (58.13)	10 (83.33)	22 (6.06)	
Hospital, n (%) 0.043				
Indiana University Hospital	231 (93.90)	12 (100)	320 (88.15)	
Parkview Hospital	15 (6.10)	0 (0)	43 (11.85)	
Charlson Comorbidity Index (CCI) Score 0.001				
CCI 0–3	206 (83.74)	8 (66.67)	260 (71.63)	
CCI 4–6	30 (12.20)	1 (8.33)	82 (22.59)	
CCI > 6	10 (4.07)	3 (25.00)	21 (5.79)	

*EndoFLIP* endoscopic functional lumen imaging probe, *POEM* per-oral esophageal myotomy

### Outcomes

#### Temporal trends in achalasia technique utilization

**Fig. 2:**
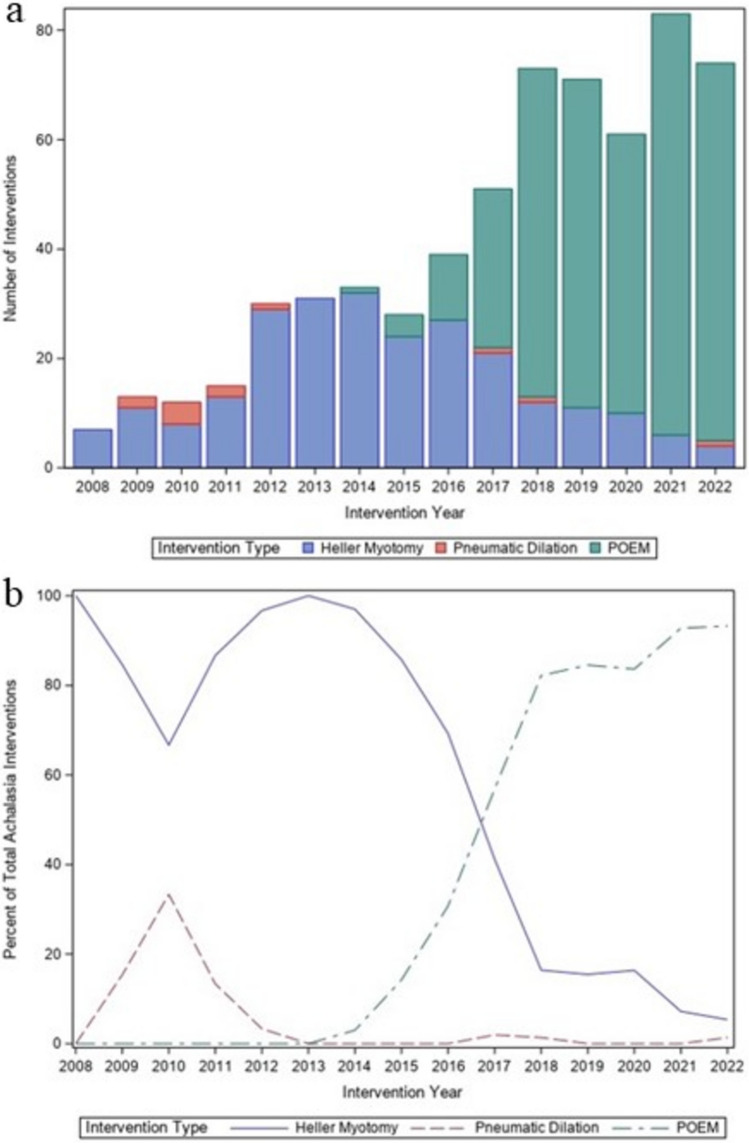
Temporal trend in the number and type of definitive achalasia procedures (**a**) and the percentage of each definitive achalasia procedure (**b**) performed in Indiana between 2008 and 2022

The year-to-year trends in frequency and percentage of overall use of each technique statewide are shown in Figs. 2a and b, respectively. The first documented POEM in the state of Indiana occurred in 2014. POEM was used once (3.03% of all statewide procedures) for the index treatment of achalasia statewide in 2014 and increased to 69 (93.24%) in 2022. The use of HM increased from 7 (100% of all statewide procedures) in 2008 to 31 (100%) in 2013, then decreased to 4 (5.41%) in 2022. The use of pneumatic dilation decreased from 9 (8.33% of all statewide procedures) from 2008 to 2013 to 3 (0.058%) from 2014 to 2022. Overall, the number of statewide interventions increased from 7 in 2008 to 74 in 2022, which equates to an increase of 5.48 procedures per year when fit to a linear model.

#### Post-intervention healthcare utilization

##### Early (≤ 30-days post-procedure)

Within 30 days of intervention, post-procedure esophagrams were more frequent after POEM (p < 0.001); the frequency of other procedures was similar between the three groups (Table 2). There was no difference in the frequency of post-procedure ICU admissions or post-discharge hospital readmissions or emergency room visits (Table 3). There were no deaths in the study cohort.

**Table 2:** Comparison of post-intervention testing in patients who underwent HM, Pneumatic Dilation, and POEM

	Heller myotomy (*n* = 246)	Pneumatic Dilation (*n* = 12)	POEM (*n* = 363)	p value
Diagnostic testing				
Early (< 30 days) post-intervention period				
pH-based studies, n (%)	0 (0.00)	0 (0.00)	1 (0.28)	1.00
Esophagram, n (%)	56 (22.76)	7 (58.33)	318 (87.60)	< 0.001
Upper endoscopy, n (%)	5 (2.03)	0 (0.00)	10 (2.75)	0.85
Related chest CT, n (%)	5 (2.03)	0 (0.00)	8 (2.20)	1.00
Esophageal manometry, n (%)	0 (0.00)	0 (0.00)	1 (0.28)	1.00
EndoFLIP, n (%)	0 (0.00)	0 (0.00)	3 (0.83)	0.32
Other modalities, n (%)	20 (8.13)	0 (0.00)	18 (4.96)	0.27
Medium-s to long-term (31 days to one year) post-intervention period				
PH-based studies, n (%)	2 (0.81)	0 (0.00)	133 (36.64)	< 0.001
Esophagram, n (%)	28 (11.38)	5 (41.67)	16 (4.41)	< 0.001
Upper endoscopy, n (%)	23 (9.35)	4 (33.33)	233 (64.19)	< 0.001
Chest CT, n (%)	5 (2.03)	1 (8.33)	0 (0.00)	0.003
Manometry, n (%)	5 (2.03)	1 (8.33)	147 (40.50)	< 0.001
EndoFLIP, n (%)	1 (0.41)	0 (0.00)	209 (57.58)	< 0.001
Other modalities, n (%)	7 (2.85)	0 (0.00)	2 (0.55)	0.064

*CT* computed tomography, *EndoFLIP* endoscopic functional lumen imaging probe, *ICU* intensive care unit, *ED* emergency department, *Botox* Botulinum toxin, *POEM* per-oral esophageal myotomy

**Table 3:** Comparison of post-intervention healthcare resource utilization in patients who underwent HM, pneumatic dilation, and POEM

	Heller myotomy (*n* = 246)	Pneumatic Dilation (*n* = 12)	POEM (*n* = 363)	p value
Early (< 30 days) post-intervention healthcare resource utilization				
ICU Stay, n (%)	8 (3.28)	0 (0.00)	3 (0.83)	0.062
Death, n (%)	0 (0.00)	0 (0.00)	0 (0.00)	
Post-discharge hospital readmission, n (%)	10 (4.07)	0 (0.00)	27 (7.44)	0.22
ED visit after index discharge, n (%)	14 (5.69)	0 (0.00)	13 (3.58)	0.36
Temporizing reintervention within a year of index intervention				
Any temporizing intervention, n (%)	6 (2.44)	0 (0.00)	†2 (0.55)	0.16
Endoscopic, non-pneumatic dilation, balloon < 30 mm, n (%)	6 (2.44)	0 (0.00)	2 (0.55)	0.16
Botox injection, n (%)	0 (0.00)	0 (0.00)	1 (0.28)	1.00
Definitive reintervention within a year of index intervention				
Any repeat intervention, n (%)	6 (2.44)	6 (50.00)	1 (0.28)	< 0.001
Laparoscopic Heller myotomy, n (%)	2 (0.81)	0 (0.00)	0 (0.00)	0.20
Open Heller myotomy, n (%)	0 (0.00)	0 (0.00)	0 (0.00)	
Pneumatic dilation, n (%)	3 (1.22)	6 (50.00)	1 (0.28)	< 0.001
POEM, n (%)	1 (0.41)	0 (0.00)	0 (0.00)	0.42

^†^One POEM patient underwent both endoscopic non-pneumatic dilation < 30 mm and Botox injection

##### Medium- to long-term (31 days to 1-year post-procedure)

Post-procedure-related pH testing (*p *< 0.001), EGD (*p* < 0.001), esophageal manometry (*p* < 0.001), and EndoFLIP (*p* < 0.001) were more common in the POEM group. Esophagrams (*p* < 0.001) and chest CT scans (*p* = 0.003) related to evaluation of achalasia were more frequent in the PD group. Within one year of index achalasia treatment, there was no difference in the frequency of the 9 temporizing interventions (Botox injection and/or small balloon dilation) performed among the three groups. However, for the 13 definitive reinterventions performed, there was a significantly higher rate of repeat interventions in the PD (50%) and HM (2.44%) groups compared to POEM (0.28%, *p* < 0.001). The above is shown in Table 3.

Adjusted outcomes comparing HRU among the three groups are shown in Table 4. Multivariate regression did not show any significant difference in early utilization of hospital services following POEM compared to HM (OR 0.87, 95% CI, 0.34–2.24, *p* = 0.78) or pneumatic dilation (OR 1.56, 95% CI, 0.07–37.49, *p* = 0.78). Within one year of intervention, diagnostic testing was more frequent after POEM compared to HM (OR 20.87, 95% CI, 10.76–40.47) but similar following POEM and PD (OR 3.25, 95% CI 0.64–16.52, *p* = 0.16). The use of temporizing procedures was similar between POEM and HM (OR 0.36, 95% CI, 0.06–2.23, *p* = 0.28) and between POEM and PD (OR 0.29, 95% CI, 0.01–8.99, *p* = 0.48). However, repeat definitive achalasia reinterventions were less frequent following POEM compared to both HM (OR 0.10, 95% CI, 0.01–0.62, *p* = 0.014) and PD (OR 0.002, 95% CI, 0.0001–0.023, *p* < 0.001).

**Table 4:** Adjusted outcomes of short- and long-term healthcare resource utilization for patients who underwent treatment with POEM compared to Heller myotomy and pneumatic dilation

Variables	POEM vs Heller myotomy		POEM vs pneumatic dilation	
	OR (95% CI)	p value	OR (95% CI)	p value
Hospital utilization within 30 days of index procedure	0.87 (0.34, 2.24)	0.78	1.56 (0.07, 37.49)	0.78
Diagnostic testing within one year of index procedure	20.87 (10.76, 40.47)	< 0.001	3.25 (0.64, 16.52)	0.16
Temporizing procedure within one year of index procedure	0.36 (0.06, 2.23)	0.28	0.29 (0.01, 8.99)	0.48
Definitive reintervention within one year of index procedure	0.10 (0.01, 0.62)	0.014	0.002 (0.0001, 0.023)	< 0.001

	POEM vs Heller myotomy	
	OR (95% CI)	P value
Sensitivity Analysis		
Hospital utilization within 30 days of index procedure	0.86 (0.33, 2.23)	0.76
Diagnostic testing within one year of index procedure	20.38 (10.47, 39.67)	< 0.001
Temporizing procedures within one year of index procedure	0.38 (0.06, 2.36)	0.30
Definitive reintervention within one year of index procedure	0.11 (0.02, 0.74)	0.024

*POEM* per-oral esophageal myotomy, *OR* odds ratio

When excluding the twelve PD patients, multivariate logistic regression modeling did not show any statistically significant difference between the POEM and HM for HRU within 30 days (OR 0.86, 95% CI, 0.33–2.23, *p* = 0.76) or temporizing procedures within 1 year (OR 0.38, 95% CI, 0.06–2.36, *p* = 0.30) following intervention (Table 4). However, POEM was associated with more frequent diagnostic testing (OR 20.38, 95% CI, 10.47–39.67, *p* < 0.001) and less frequent definitive reintervention (OR 0.11, 95% CI, 0.02–0.74, *p *= 0.024) compared to the HM group.

#### Secondary outcomes

##### Safety profile comparison

The frequency and severity grade of AEs for each intervention are outlined in Table 5. Periprocedural AEs were similar between the HM (16.67%), POEM (13.22%), and PD (0%) groups (*p* = 0.22). Similarly, the severity of these events was similar for each group. There was no recorded mortality.

**Table 5:** Frequency and severity of adverse events for patients treated with Heller myotomy, pneumatic dilation, and POEM

	Heller myotomy (*n* = 246)	Pneumatic Dilation (*n* = 12)	POEM (*n* = 363)	p value
Periprocedural adverse events, n (%)				
Any adverse event	41 (16.67)	0 (0.00)	48 (13.22)	0.22
Bleeding requiring transfusion or interventions	1 (0.41)	0 (0.00)	1 (0.28)	1.00
Perforation	7 (2.85)	0 (0.00)	4 (1.10)	0.30
Pneumothorax requiring intervention	2 (0.81)	0 (0.00)	2 (0.55)	1.00
Other non-infectious pulmonary/respiratory issue	4 (1.63)	0 (0.00)	9 (2.48)	0.67
Myocardial infarction	0 (0.00)	0 (0.00)	0 (0.00)	
Other non-infectious cardiac issue	6 (2.44)	0 (0.00)	8 (2.20)	1.00
Cerebrovascular Event	0 (0.00)	0 (0.00)	0 (0.00)	
Infection	10 (4.07)	0 (0.00)	6 (1.65)	0.19
Other	20 (8.13)	0 (0.00)	27 (7.44)	0.81
Adverse event Severity				
Grade				0.20
Grade 1	17 (41.46)	0 (0.00)	28 (58.33)	
Grade II	11 (26.83)	0 (0.00)	7 (14.58)	
Grade III	8 (19.51)	0 (0.00)	11 (22.92)	
Grade IV	5 (12.20)	0 (0.00)	2 (4.17)	
Grade V – (Death)	0 (0.00)	0 (0.00)	0 (0.00)	

Adverse event severity graded using the Clavien–Dindo system for Heller myotomy and the AGREE (adverse events in gastrointestinal endoscopy) classification for POEM (per-oral esophageal myotomy) and PD (pneumatic dilation)

## Discussion

This 14-year retrospective statewide cohort study confirmed a shift in achalasia treatment, with POEM becoming the dominant index procedure since its adoption in 2014, largely replacing HM. POEM use increased from 3% in 2014 to 93% in 2022, while HM decreased from 100% in 2013 to 5% in 2022. These findings align with recent retrospective studies of United States commercial insurance databases documenting declining hospitalizations for HM but a rise in admissions for POEM[5, 11]. Kilani et al.[6] found that hospital admissions for achalasia following POEM increased from 6 to 10% while those following HM decreased from 49 to 41% from 2016 to 2020. Lois et al.[7] reported a 19-fold increase in use of POEM and decreased use of LHM and PD between 2010 and 2017. Our study identified a more dramatic rise and decline in the state of Indiana in the use of POEM and HM, respectively.

The rapid adoption of POEM likely stems from factors including increased endoscopist training, favorable randomized trial result [3, 4], professional society guideline endorsements [12–14], and establishment of a specific CPT code in 2022. Furthermore, POEM is an incisionless endoscopic procedure and may be preferred in patients with increased comorbidities. Our data support this, showing a higher proportion of POEM patients had CCI scores of 4–6 compared to HM patients (22.6% vs 12.2%), while HM patients more frequently had CCI scores of 0–3 (83.7% vs 71.6%). Similar findings regarding higher CCIs in POEM patients have been reported elsewhere [11].

Despite differences in CCI scores, we found that the frequency and severity of adverse events were similar between the three interventions for achalasia. Severe AEs (Grade III-V) were reported in 5.28%, 0%, and 3.58% of HM, PD, and POEM groups, respectively. While some studies have reported similar frequency of overall and severe adverse events for these procedures [4–6], others have reported higher AEs with POEM compared to HM [15].

Our study highlights some differences in the frequency of preoperative and post-operative testing among the three interventions. In our cohort, the frequency of a preoperative EGD, esophageal manometry, and EndoFLIP testing was higher for the POEM group but preoperative esophagram was more common in patients undergoing HM. One month or more after intervention, the POEM group was more likely to undergo diagnostic testing including esophageal pH testing, EGD, esophageal manometry, and EndoFLIP testing. The increased use of post-operative testing in the POEM group remained after sensitivity analysis with elimination of the small PD cohort in our study. This increased testing reflects initial protocol-driven clinical efforts during early adoption of POEM at both institutions to understand the efficacy, safety, adverse events (particularly reflux), and outcomes of the procedure. These routine post-procedure tests likely contribute to higher costs as noted in other studies [6]. However, hospital resources for testing in all POEM patients are limited, and recent evidence suggests routine post-POEM esophagrams and delayed surveillance testing may not be necessary for all patients [16–18]. Additionally, same-day discharge after POEM is feasible, potentially allowing for reduced HRU and cost reduction. We also found that when compared to PD and HM, POEM was associated with decreased frequency of definitive reintervention within one year of index treatment. These findings remained consistent between POEM and HM after sensitivity analysis. Thus, we surmise that decreased requirement for post-operative testing and disease reintervention may decrease the previously described increased costs of POEM relative to HM[6] and is supported by findings by Conte et al.[19].

PD utilization was low (n = 12, 1.9%) throughout the study period, likely reflecting limited local expertise or training among endoscopists. This precludes robust comparisons involving this modality. To mitigate this limitation, however we excluded this cohort and performed a sensitivity analysis using multivariate logistic regression modeling to directly compare the various metrics of HRU between the POEM and HM groups.

Our study has some limitations. First, while the Indiana Health Information Exchange (IHIE) statewide database used for this study captures nearly all Indiana healthcare data, some cases might be missed in this analysis. Second, reliance on ICD/CPT codes may lead to misclassification of diagnoses or procedures if entered incorrectly by healthcare providers. Third, the small PD cohort limits generalizable comparisons of this technique with POEM and HM. Fourth, study outcomes are limited to a one-year follow-up period which may be insufficient to evaluate long-term durability of these treatment strategies. Fifth, healthcare utilization was analyzed but a cost analysis was not performed. Finally, this study was limited to two tertiary care centers in the state of Indiana, which may limit generalizability to a broader national and international practice.

In conclusion, our retrospective 14-year study of the state of Indiana identified an increased utilization of POEM and decreased utilization of HM for index definitive achalasia treatment. In our cohort, POEM was associated with more frequent post-procedure HRU, lower odds of disease reintervention, and similar rates of adverse events compared to HM or PD.
